# Improving medication safety: Development and impact of a multivariate model-based strategy to target high-risk patients

**DOI:** 10.1371/journal.pone.0171995

**Published:** 2017-02-13

**Authors:** Tri-Long Nguyen, Géraldine Leguelinel-Blache, Jean-Marie Kinowski, Clarisse Roux-Marson, Marion Rougier, Jessica Spence, Yannick Le Manach, Paul Landais

**Affiliations:** 1 Department of Pharmacy, Nîmes University Hospital, Nîmes, France; 2 Laboratory of Biostatistics, Epidemiology, Clinical Research and Health Economics, University Institute of Clinical Research, Montpellier University, Montpellier, France; 3 Departments of Anesthesia, Michael DeGroote School of Medicine, Faculty of Health Sciences, McMaster University, Hamilton, Ontario, Canada; 4 Population Health Research Institute, David Braley Cardiac, Vascular and Stroke Research Institute, Perioperative Medicine and Surgical Research Unit, Hamilton, Ontario, Canada; 5 Department of General Medicine, Nîmes University Hospital, Nîmes, France; 6 Department of Biostatistics, Epidemiology, Clinical Research and Health Economics, Nîmes University Hospital, Nîmes, France; Universita degli Studi di Perugia, ITALY

## Abstract

**Background:**

Preventive strategies to reduce clinically significant medication errors (MEs), such as medication review, are often limited by human resources. Identifying high-risk patients to allow for appropriate resource allocation is of the utmost importance. To this end, we developed a predictive model to identify high-risk patients and assessed its impact on clinical decision-making.

**Methods:**

From March 1^st^ to April 31^st^ 2014, we conducted a prospective cohort study on adult inpatients of a 1,644-bed University Hospital Centre. After a clinical evaluation of identified MEs, we fitted and internally validated a multivariate logistic model predicting their occurrence. Through 5,000 simulated randomized controlled trials, we compared two clinical decision pathways for intervention: one supported by our model and one based on the criterion of age.

**Results:**

Among 1,408 patients, 365 (25.9%) experienced at least one clinically significant ME. Eleven variables were identified using multivariable logistic regression and used to build a predictive model which demonstrated fair performance (c-statistic: 0.72). Major predictors were age and number of prescribed drugs. When compared with a decision to treat based on the criterion of age, our model enhanced the interception of potential adverse drug events by 17.5%, with a number needed to treat of 6 patients.

**Conclusion:**

We developed and tested a model predicting the occurrence of clinically significant MEs. Preliminary results suggest that its implementation into clinical practice could be used to focus interventions on high-risk patients. This must be confirmed on an independent set of patients and evaluated through a real clinical impact study.

## Introduction

Drug-related problems result in substantial medical and economic burden, with an estimated cost of $177.4 billion per year [1]. They are commonly defined as “events or circumstances involving drug therapy that actually or potentially interfere with desired health outcomes” [2]. These include both adverse drug events, which result in actual harm, and clinically significant medication errors (called “potential adverse drug events”), which have the potential to cause harm in absence of correction. Review of the literature suggests that medication errors (MEs) are significantly common, occurring in up to 67% of hospitalized patients [3]. Given their preventability, frequency, and potential association with adverse patient outcomes [4–6], strategies for reducing clinically significant MEs are of the utmost importance.

Several studies have highlighted the role of pharmacist-led medication review for preventing potential adverse drug events [6–16]. By closely collaborating with each other, physician and pharmacist complement one another and attempt to address patient needs. This inter-professional intervention, done in association with the medical team, consists of optimization of medication regimens after home medication reconciliation at admission, pharmaceutical analysis and review of currently prescribed drugs [6–16]. The process is very time consuming and, because of competing clinical demands, review of all inpatients by a clinical pharmacist is usually not possible. As a consequence, those at increased risk of potential adverse drug events need to be identified and considered a priority for evaluation.

Targeting “high-risk” patients might be a key aspect of successful interventions [6, 16]. Nevertheless, commonly chosen criteria, such as age or number of medications [16], often fail to identify these patients [6]. Instead of considering a single predictor strategy, we propose a multivariate model-based strategy to detect these high-risk patients. Several approaches to the prediction of actual or potential adverse drug events are described in the literature [17–21], though they vary in terms of definition. While some focus on a subcategory [17–20], others are specific to certain age groups or diagnostic populations [17, 19, 20]. Rather than focus on a specific subgroup, our approach aims to develop a generalizable tool for daily practice that includes non-invasive predictors of avoidable outcomes that are readily available at the time of hospital admission.

We report the derivation of a model predicting in-hospital significant medication errors (PRISMOR), according to the Transparent Reporting of a multivariable prediction model for Individual Prognosis Or Diagnosis (TRIPOD) statement [22, 23] and assess its clinical impact on decision-making compared to a strategy based on age.

## Methods

Data collection for the study was approved by the French National Commission of Information Technology and Civil Liberties (CNIL, *Commission Nationale de l’Informatique et des Libertés*; agreement No. 1738666) and the Institutional Review Board of the Nîmes University Hospital Centre, which waived the need for written informed consent (No. IRB 14/01-03). Patient information were de-identified prior to analysis.

### Design

We conducted a prospective cohort study in the Nimes University Hospital Centre from March 1^st^ to April 31^st^, 2014, targeting a sample size that would result in 20 events per variable [24, 25]. Based on a recent systematic review reporting 45% of patients with at least one clinically significant ME [6], we estimated a required sample size of 1,000 patients. All patients over 17 years old admitted to hospital during the study period were included. Patients were followed from admission to discharge. During admission, all prescribed medications were reviewed by one of 15 clinical pharmacists, all of whom were trained in standardized medication reconciliation, prescription analysis and ME reporting.

### Endpoint and predictors

The primary outcome was the occurrence of at least one clinically significant ME at any time during in-hospital stay. MEs were defined as either unintentional medication discrepancies (*i*.*e*. involuntary divergence between admission prescription and best possible medication history obtained from a minimum of 3 sources of information) or prescribing errors (*i*.*e*. non-adherence to recognized clinical prescribing guidelines). Given their more imprecise nature, two clinicians (a physician and a pharmacist) independently reviewed all MEs using the method described by Quélennec *et al*. [26], a European adaptation of the National Coordinating Council for Medication Error Reporting and Prevention (NCC MERP) Index [27]. Potential clinical impact of MEs was rated regarding patient’s clinical characteristics, medications, biological data and number of MEs. MEs were classified into 3 categories: “without potential harm (NCC MERP category C)”, “requiring potentially monitoring or intervention to preclude harm (NCC MERP category D)” and “with potential harm (NCC MERP category E and above)”. Disagreements were resolved by a third evaluator. MEs without potential harm were not considered as events of interest in the primary analysis.

For risk prediction, we extracted patient-, hospitalisation-, and prescription-related covariates from the electronic medical record. Specifically, age, sex, admission details, admission type (medical or surgical), hospital admission within the preceding 30 days, hour (day or night) and day of admission (weekday or week-end), number of prescribed drugs and drug classes (according to the Anatomical Therapeutic Chemical (ATC) classification system). We also documented whether a current treatment had been initiated before admission and whether a best possible medication history had been obtained before the admission medication order.

### Statistical analysis

Univariate analyses were performed using Wilcoxon-Mann-Whitney test for continuous variables and Chi-square test for binary variables. Relations between drug ATC classes and the endpoint were explored using association rules mining. This method is useful to detect co-occurrence of an endpoint with multiple binary covariates in the absence of an *a priori* hypothesis [28]. The algorithm was applied assuming a minimal support (*i*.*e*. minimal probability of co-occurrence) of 5% and a minimal confidence (*i*.*e*. minimal probability of endpoint occurrence given the covariates) of 10%. ATC classes found to be significant using association rules were then included in logistic regression. Variables with a *P* less than 0.50 were considered eligible for inclusion in the multivariate model.

For the multivariate model development, a logistic regression was performed. Because a strict selection criterion would lead to discarding informative variables, the alpha risk threshold (*P* <0.30) was intentionally set as large in order to provide reliable estimates and performances [25, 29, 30]. Multivariable fractional polynomials analysis was conducted to take into account non-linearity of continuous variables. The apparent performance of the model was described using c-statistic and a calibration curve. Corrected performances were reported after bootstrapping (b = 500) for internal validation [24, 31, 32].

**Fig 1:**
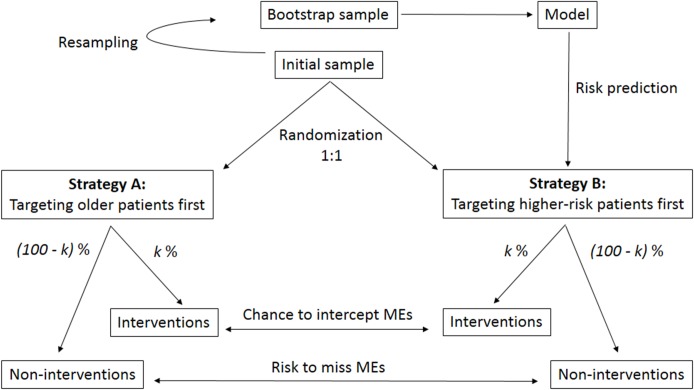
Simulated randomized controlled trial comparing two decision-making strategies for intervention to reduce medication errors. The analysis was reiterated 1,000 times within 5 scenarios, within each the intervention coverage *k* was fixed at 10%, 30%, 50%, 70% and 90%.

In order to evaluate the impact of implementing our predictive model into clinical practice we compared two strategies for pharmaceutical intervention. The first involved clinical decision-making based on age, whereas the second was based on the PRISMOR model. To this end, we conducted a series of simulated randomized controlled trials (RCTs), which allowed us to test our model under the conditions of variable pharmacist staff coverage (Fig 1). The endpoints of these trials were the interception of at least one harmful ME during the intervention and the failure to identify at least one harmful ME in the absence of intervention. We simulated the situation, in which such a model (training model) would be applied on an external sample (testing sample) [31, 32]. After fitting a similar predictive model on a bootstrap sample (training model), we randomly assigned patients from our initial sample (testing sample) into one of two groups in a 1:1 ratio. In group A (control), older patients were identified as a priority for pharmacist review whereas, in group B, patient priority was determined by the predicted probability of ME, provided by the training model. This simulation was repeated 1,000 times within five scenarios, within each the pharmacy intervention coverage was set to 10%, 30%, 50%, 70% or 90% of patients.

Analyses were conducted with R 2.15.2 (R Development Core Team. R Foundation for Statistical Computing, Vienna, Austria).

## Results

A total of 1,408 patients admitted to 21 hospital units (4 surgical and 17 medical) were included. Median age was 68 years [IQR: 57–80] and 738 (52.4%) were males. Median length of stay was 4 nights [IQR: 2–8]. Baseline characteristics are reported in Table 1.

**Table 1:** Baseline patient characteristics.

		Occurrence of ME(s)		Univariate P*
** **	** **	**No (%)**	**Yes (%)**	
Sex	Male	560 (75.9)	178 (24.1)	0.119
	Female	483 (72.1)	187 (27.9)	
Age (year old)		Median: 66	Median: 72	< 0.001
Number of prescribed drugs		Median: 1	Median: 4	< 0.001
Treatment initiated before entrance	No	104 (94.5)	6 (5.5)	< 0.001
	Yes	939 (72.3)	359 (27.7)	
Best possible medication history	No	426 (67.0)	210 (33.0)	< 0.001
	Yes	617 (79.9)	155 (20.1)	
Previous hospitalization within 30 days	No	912 (73.4)	331 (26.6)	0.118
	Yes	131 (79.4)	34 (20.6)	
Transfer from other unit within 72 hours	No	1 023 (74.5)	351 (25.5)	0.063
	Yes	20 (58.8)	14 (41.2)	
Admission from emergency room	No	928 (76.1)	292 (23.9)	< 0.001
	Yes	115 (61.2)	73 (38.8)	
Admission from an outside institution	No	1 017 (73.9)	360 (26.1)	0.293
	Yes	26 (83.9)	5 (16.1)	
Admission time	Day (9 AM to 8 PM)	863 (74.5)	295 (25.5)	0.455
	Night (8 PM to 9 AM)	180 (72.0)	70 (28.0)	
Admission day	Weekday	952 (74.5)	326 (25.5)	0.313
	Week-end (or holiday)	91 (70.0)	39 (30.0)	
Type of hospital admission	Medical	794 (75.4)	259 (24.6)	0.059
	Surgical	249 (70.1)	106 (29.9)	

* Wilcoxon-Mann-Whitney test for continuous variables, Chi square test for binary variables. ME, medication error.

**Fig 2:**
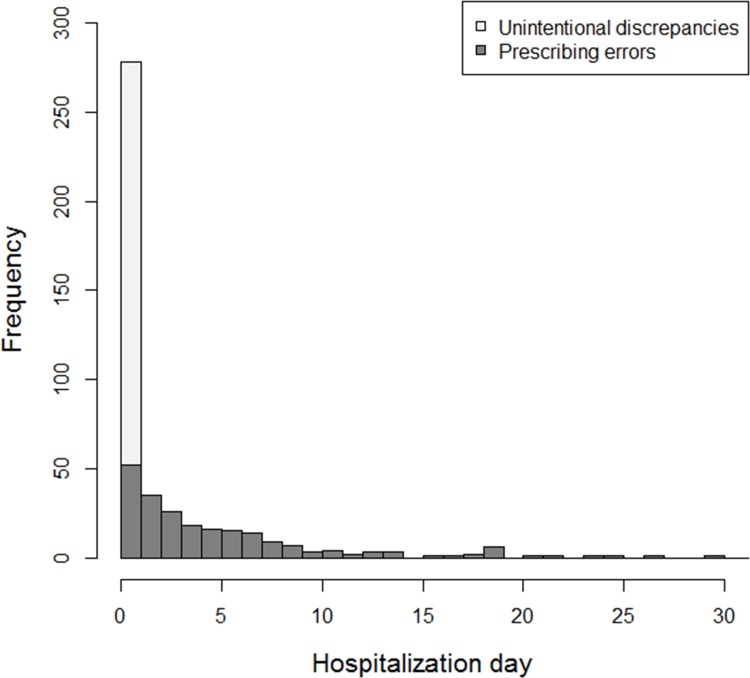
Distribution of clinically significant medication errors according to hospitalization day.

During the study period, pharmacists identified 609 MEs. Of these, 475 were considered as events of interest due to their association with harm or potential harm (Table 2). Most occurred on the first day of hospitalization (Fig 2), with a majority of unintentional discrepancies resulting from inaccurate information about current medications, which were corrected by physician after notification. Three MEs actually led to adverse drug events, due to a failure of correction at time (Table 2). During their stay, 365 (25.9%) of patients included experienced at least one clinically significant ME. Occurrence of ME was associated with 5 ATC classes: antithrombotic agents (B01), antibacterial agents for systemic use (J01), psycholeptics (N05), blood substitutes and perfusion solutions (B05) and analgesics (N02).

**Table 2:** Description of reported medication errors, with illustrative examples.

**MEs leading to actual harm: 3 (0.5%)**
Respiratory depression after Midazolam overdose (requiring Flumazenil)
Bleeding and hematoma secondary to non-adaptation of antivitamin K therapy
Cardiogenic pulmonary edema due to the unintentional omission of diuretic and beta-blocker medications (patient with chronic heart failure)
**Potential harmful MEs (NCC MERP category E and above): 157 (25.8%)**
Underdose of LMWH (preventive dose instead of curative dose, patient admitted for atrial fibrillation)
Unintentional omission of Levetiracetam (patient with previous status epilepticus)
Unintentional omission of beta-blocker, statin, ACE inhibitor and Metformin (patient with previous acute coronary syndrome and diabetes mellitus type 2)
Unintentional dose reduction of Flecainide (patient with atrioventricular nodal reentrant tachycardia)
Unintentional double-dosing of Digoxin (patient with glomerular filtration rate of 36 mL/min)
**MEs requiring potentially monitoring or intervention to preclude harm (NCC MERP category D): 315 (51.7%)**
Co-prescription of two angiotensin-II receptor antagonists
Inappropriate dose regimen of Gentamicin (three times a day instead of once a day)
Unintentional double-dosing of Bisoprolol (patient with atrioventricular block)
Unintentional dose reduction of Lithium (patient with bipolar disorder)
Unintentional omission of Methotrexate (patient with rheumatoid arthritis)
Non harmful MEs* (NCC MERP category C): 134 (22.0%)
Unintentional omission of Zolpidem
Unintentional addition of Esomeprazole (no indication)
Unintentional dose augmentation of Pravastatin
Inappropriate dose regimen of Budesonide and Formoterol (once a day instead of twice a day)
Double prescription of Macrogol

* Non harmful MEs were not considered as being part of the 475 events of interest. ME, medication error.

For clinically significant ME prediction, we fitted a multivariate logistic model composed of 11 predictors (Table 3). ME was significantly associated with the presence of a current treatment initiated before admission (OR = 5.64, 95% CI: 2.38–13.36, P< 0.001), number of prescribed drugs (OR = 1.16 per drug, 95% CI: 1.10–1.23, P< 0.001) and increasing age (P = 0.005 and 0.010 for quadratic and cubic terms, respectively). The relationship between age and ME risk followed an “n-shaped” curve (S1 Fig) indicating a maximal risk around 75 years old (OR = 4.26, 95% CI: 3.56–5.09). Risk of ME decreased significantly when medication history was completed prior to admission medication orders (OR = 0.50, 95% CI: 0.37–0.67, P< 0.001). Three predictors had associations with ME that almost achieved statistical significance: admission to a surgical ward (OR = 1.36, 95% CI: 1.00–1.87, P = 0.061), admission to hospital within the last month (OR = 0.68, 95% CI: 0.44–1.04, P = 0.067) and prescription of psycholeptic medication (ATC class N05) (OR = 1.39, 95% CI: 0.96–2.02, P = 0.084). As suggested by Steyerberg *et al*. [29, 30], other informative predictors were also included in the final model despite their non-significance.

**Table 3:** Multivariate model predicting in-hospital significant medication errors (PRISMOR).

		Corrected log-odds ratio*	Estimated odds ratio [95% CI]	*P*
*Constant*	** **	-3.83	0.02	< 0.001
(Age/100)^2^		7.07	2 079.74 [10.26; 421 510.51]	0.005
(Age/100)^3^		-6.26	0.00 [0.00; 0.20]	0.010
Number of prescribed drugs		0.14	1.16 [1.10; 1.23]	< 0.001
Treatment initiated before admission	No	0	1.00	
	Yes	1.60	5.64 [2.38; 13.36]	< 0.001
Best possible medication history available	No	0	1.00	
	Yes	-0.64	0.50 [0.37; 0.67]	< 0.001
Psycholeptics	No	0	1.00	
	Yes	0.31	1.39 [0.96; 2.02]	0.084
Blood substitutes and perfusion solutions	No	0	1.00	
	Yes	-0.16	0.84 [0.62; 1.15]	0.295
Type of hospital admission	Medical	0	1.00	
	Surgical	0.29	1.36 [1.00; 1.87]	0.061
Hospital admission within previous 30 days	No	0	1.00	
	Yes	-0.36	0.68 [0.44; 1.04]	0.067
Admission from emergency room	No	0	1.00	
	Yes	0.27	1.34 [0.92; 1.94]	0.123
Admission time	Day	0	1.00	
	Night	-0.18	0.83 [0.58; 1.18]	0.296
Admission from an outside institution	No	0	1.00	
	Yes	-0.51	0.58 [0.21; 1.60]	0.299

* Original estimated log-odds ratios were corrected by a uniform shrinkage factor equal to 0.926. If *W*_*i*_ and $\log\;{odds\;ratio}_{W_{i}}$ denote each variable and its corresponding log-odds ratio, respectively, the individual predicted probability of significant medication error (ME) is calculated as:

$\hat{p}(ME)={\left\{1+exp\;[-(-3.83+\sum W_{i}∙\log\;{odds\;ratio}_{W_{i}})]\right\}}^{-1}$

Analysis of apparent performance revealed fair discrimination (c-statistic: 0.718, 95% CI: 0.689–0.748) and good calibration (intercept equal to 0 and a slope equal to 1). The local regression curve of calibration indicated a slight over-estimation of high probabilities (S2 Fig). After bootstrapping, corrected discrimination remained fair (c-statistic: 0.707) and corrected calibration suggested slight over-fitting (corrected intercept: -0.069 and corrected slope: 0.926). Regression coefficients were therefore corrected by a uniform shrinkage factor of 0.926 (Table 3).

**Fig 3:**
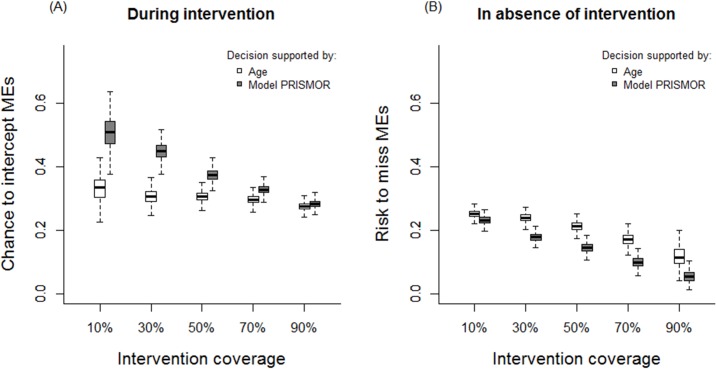
Comparison of two strategies to focus interventions on high-risk patients: decision-making supported by the predictive model *versus* decision-making based on age.

RCT simulations (n = 5,000) demonstrated the effect of the PRISMOR model on the identification and interception of MEs when compared to identification based on age (Fig 3). In all clinical coverage scenarios, clinical pharmacists were more likely to identify MEs when the PRISMOR model was used to target high-risk patients. This effect was strongest in the 10% coverage scenario, with 17.5% mean improvement over age-based selection (statistical significance *P* <0.05 in 83.2% of simulations) and an absolute probability of ME interception of 50.6%. This corresponds to a number needed to treat of 6 to avoid at least one harmful ME and, when compared to the absence of clinical pharmacist evaluation, a number needed to treat of 2. Conversely, the simulation demonstrated that by using our prediction model, clinicians would have missed fewer MEs, with an effect that increased with clinical coverage. Similar analysis was conducted to compare the decision-making supported by our model *versus* the one based on the number of medications (S3 Fig). Although the enhancement in intercepting potential adverse drug events is more limited (mean improvement of 5.4% in the 10% coverage scenario), using our model as decision-support reduced the risk to miss clinical significant MEs in absence of intervention (absolute risk difference of -9.9%, 1 ME avoided every 10 decisions in the 90% coverage scenario).

## Discussion

The incidence [3, 4, 6, 13, 33, 34] and timing [3, 4, 34, 35] of MEs in our sample is consistent with that seen in previous studies. This literature emphasizes the importance of implementing a strategy to prevent MEs early in hospitalization, ideally prior to admission medication prescription. Using predictors identified within our sample, we developed and tested a model to inform such a strategy. As expected, age and number of medications were significant predictors of MEs. Additionally, the availability of accurate information about current medications and doses prior to prescription substantially decreased the risk of MEs. In particular, a best possible medication history could have been obtained by other clinicians. As it is usually the same as that listed in the discharge summary from any recent hospitalization, hospitalization within the previous 30 days was associated with lower risk of MEs. To inform clinical triage of patients, we evaluated the potential of broadly-defined MEs to cause harm. We chose to develop a generalizable predictive model that could be implemented in everyday practice to intercept avoidable outcomes. This model was reported using international standards [22, 23] and assessed for robustness using resampling methods.

The randomized experiments showed that targeting high-risk patients with our model had the potential to substantially improve the efficiency of pharmaceutical intervention. Implementing our model in practice could enhance the identification and interception of potentially adverse drug events, a result that was consistent across levels of clinical pharmacist coverage. In all scenarios, results suggest that our model may assist clinicians to select patients who are more likely to benefit from pharmaceutical intervention. In situations of limited pharmacy coverage, our model would theoretically enable clinicians to intercept a greater number of clinically significant MEs by intervening whereas, in high coverage scenarios, fewer MEs would be missed in those patients not undergoing pharmacy review. This trend is not surprising, since greater clinical pharmacy coverage would result in a greater proportion of patients who receive intervention, including higher-risk subjects. When complete coverage is provided, targeting high-risk patients would not improve ME interception, as pharmacists already review all subjects. In contrast, focusing interventions on high-risk patients might be essential in the setting of resource limitations. Using our model-based strategy, a new clinical pharmacy service covering 10% of inpatients would avoid a potential adverse drug event every two interventions.

A small number of studies have previously reported statistical models developed to predict medication errors [17–21]. Though apparent discriminations were comparable to ours [17–21], our study improves on these through its transparent reporting of calibration and optimism [22, 23]. More importantly, we assessed the potential impact of our tool on clinical practice. In addition, because we did not restrict our study to a specific age range or field of medicine [17, 19, 20], it can be generalized to both medical and surgical inpatient settings.

Previous studies have restricted their studies to actual adverse events [17, 19, 20]; we chose to be more inclusive, including both actual and potential adverse events. As our study was based on observational (i.e., “real-life”) data, medication reviews were being conducted by clinical pharmacists as part of routine clinical practice. As such, when they were identified, mitigating action was taken to prevent medication errors which could cause harm. Had we not taken them into account, our model would have underestimated the incidence of the outcome.

Several limitations to our study need to be considered. Our model does not take into account biological markers [19] or diagnostic category [21]. Given that data consistently demonstrates that the highest risk of potential adverse drug events is on the first day admitted to hospital, we chose to exclude these potential predictors, as we felt that the most clinically valuable model would be limited to predictors that are readily available at the time of admission to hospital. Though patient comorbidities are potential predictors, the size of our derivation set limited the inclusion of these variables in the model. Having added them as independent predictors would almost certainly have resulted in over-fitting, a recognized problem to which models derived from small cohorts are prone [36–38]. Summarizing indices, such as Charlson’s index or Elixhauser’s index, would have been inappropriate given that they were developed to predict mortality, an outcome which is very different from ours [39, 40]. Moreover, recent studies have suggested that these indices have relatively poor discrimination and calibration in their prediction of nonfatal outcomes [41, 42]. Diagnoses could have been grouped in other ways; we felt it was impractical with regard to our model, however. Indeed, comorbidities were recorded in only 723 (51.3%) patient discharge summaries, which raised a real concern for the usability of a model including these predictors. In case of no recorded information (*i*.*e*. no input value), the patient’s probability of outcome would no longer be computable using the model formula.

In spite of including an appropriate number of variables for the number of outcomes [24, 25], we had to shrink initial estimates to address over-fitting [38]. That being said, bootstrap-corrected models developed based on values of 20 events per variable tend to demonstrate similar performance in validation studies [24]. Finally, our impact analysis is limited by the assumption that reducing potential adverse events would result in reduction of actual outcomes. Our preliminary results must therefore be confirmed on an independent set of patients, ideally from other hospital centers, and the usability of our predictive tool must be evaluated through a clinical impact study.

In conclusion, we developed and internally validated a model to predict potential adverse drug events as a strategy to improve pharmacist human resource allocation and subsequent patient safety. Once external validation and evaluation of concrete clinical outcomes takes place, its incorporation into clinical practice could potentially allow institutions to identify at-risk patients at their time of arrival to hospital, thus allowing for efficient, patient-specific allocation of clinical pharmacy services.

## Supporting information

S1 Fig Age effect on drug-related problem risk. Age effect (solid line) and 95% confidence interval (dotted lines) were estimated by multivariable fractional polynomials analysis.
(TIF)

S2 Fig Model calibration curve. (TIF)

S3 Fig Comparison of two strategies to focus interventions on high-risk patients: decision-making supported by predictive model *versus* decision-making based on number of medications (1,000 simulated randomized controlled trials for each coverage value). (TIF)
