# Supplementary figures and images for: Improving medication safety: Development and impact of a multivariate model-based strategy to target high-risk patients
Source: PLoS One. 2017 Feb 13;12(2):e0171995. doi: 10.1371/journal.pone.0171995 (PMC5305217; doi:10.1371/journal.pone.0171995)

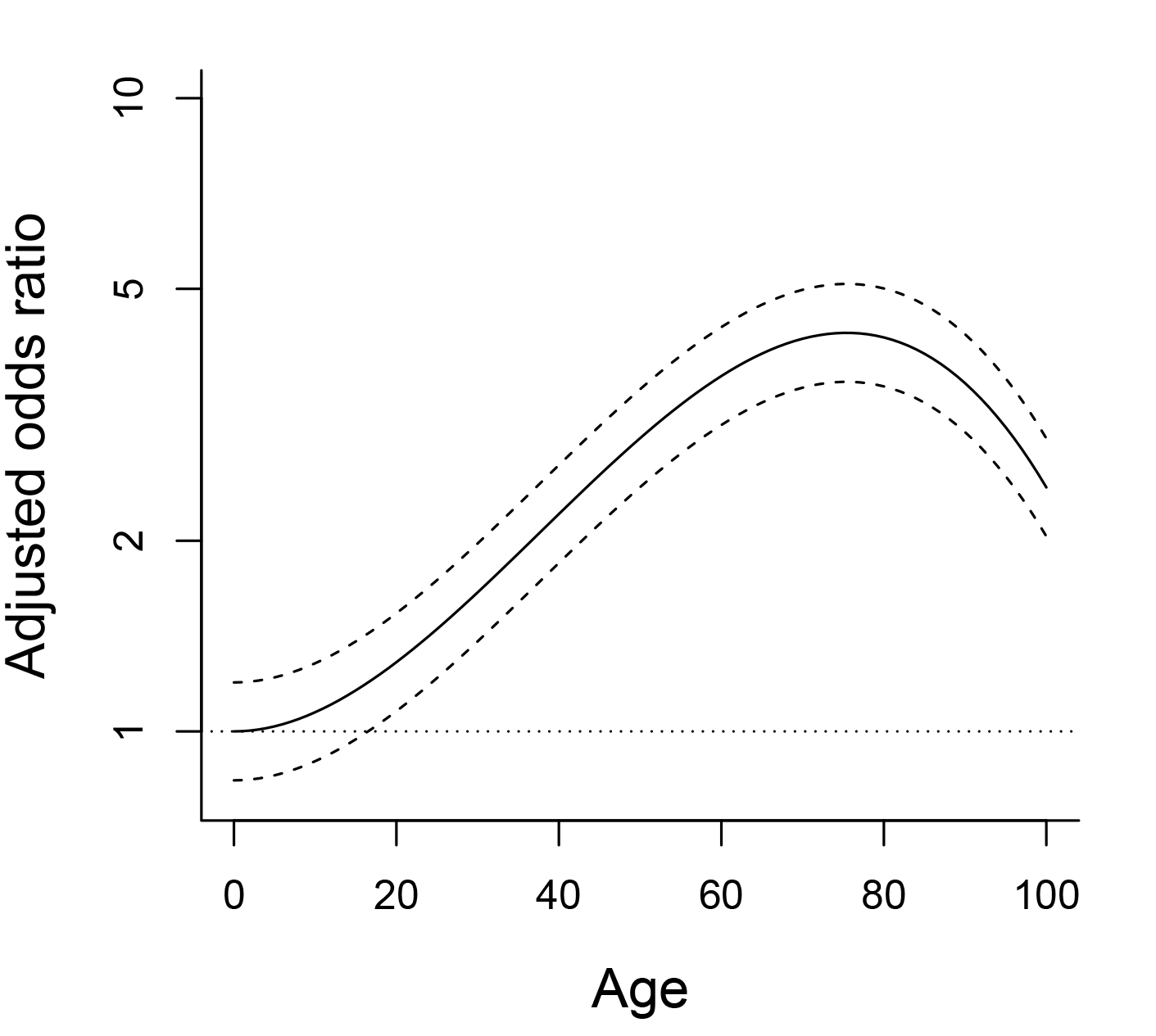

Supplement: S1 Fig — Age effect (solid line) and 95% confidence interval (dotted lines) were estimated by multivariable fractional polynomials analysis. (TIF) [file pone.0171995.s001.tif]

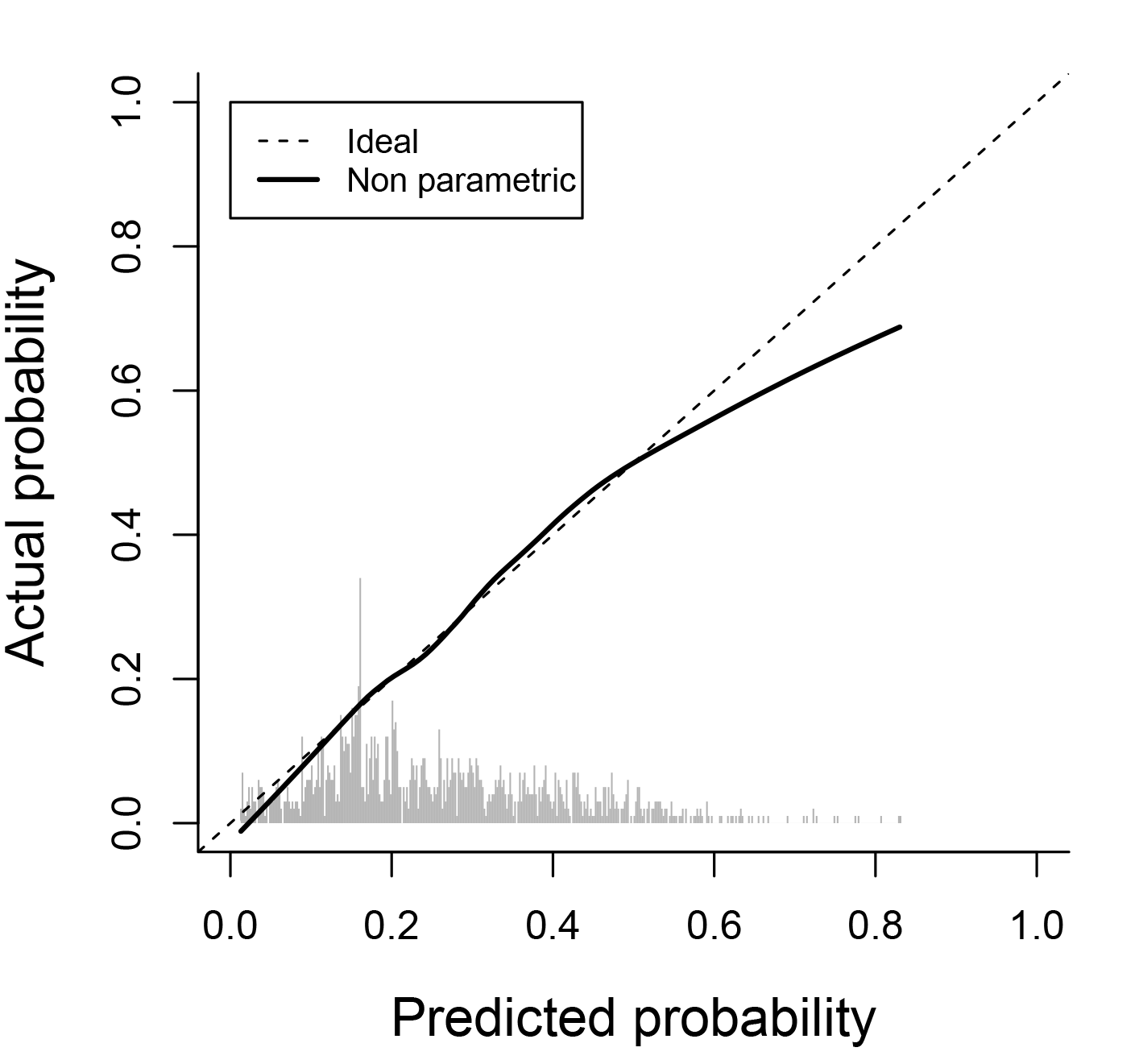

Supplement: S2 Fig — (TIF) [file pone.0171995.s002.tif]

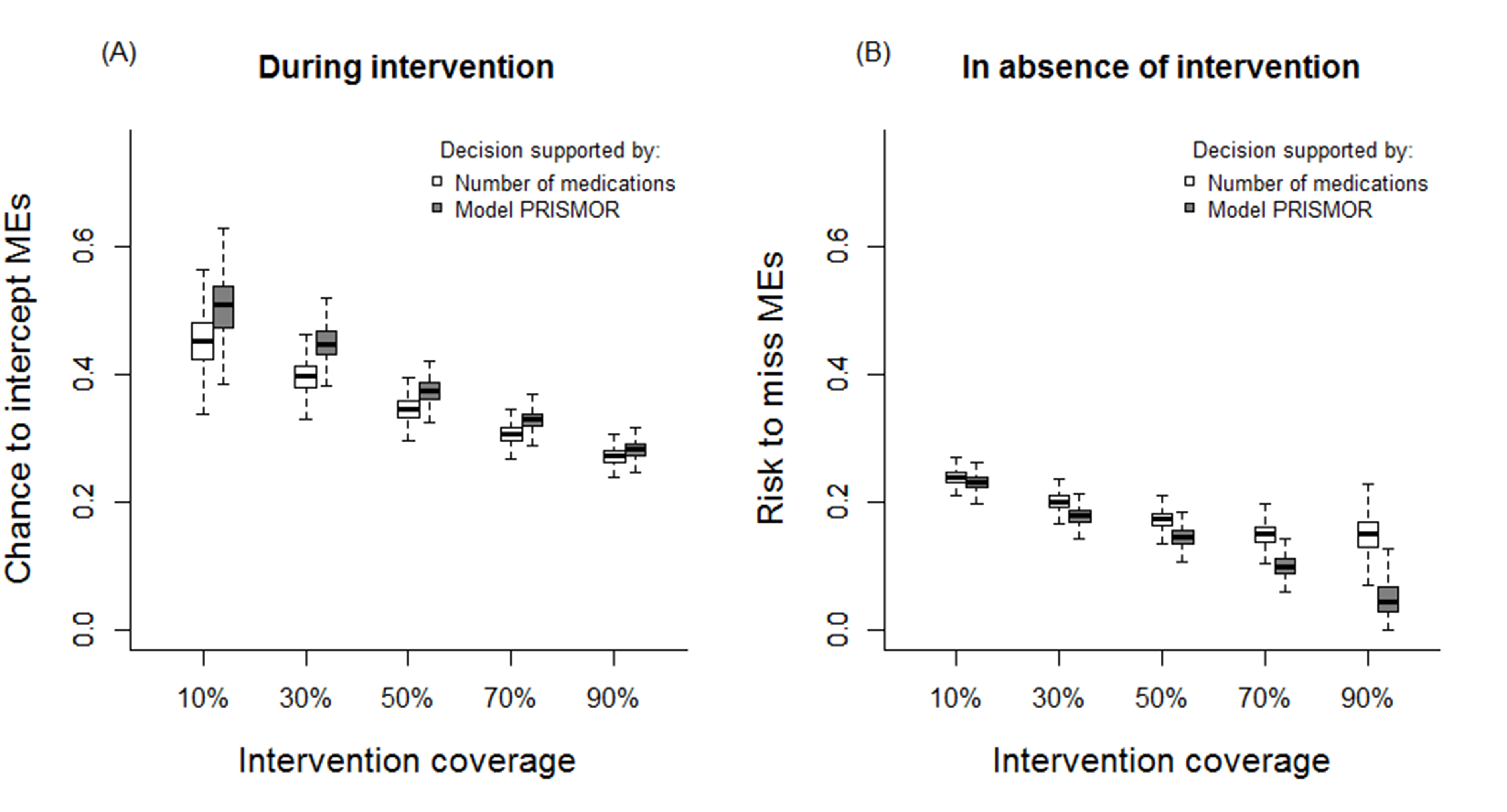

Supplement: S3 Fig — (TIF) [file pone.0171995.s003.tif]
